# Acalculous Cholecystitis: The Unexpected Mask of De Novo Heart Failure

**DOI:** 10.1002/ccr3.70324

**Published:** 2025-03-19

**Authors:** Mhd Baraa Habib, Maram Albandak, Mhd Husam Osman, Shahem Abbarh, Bisher Sawaf, Yaseen Alastal, Abdulrahman Arabi

**Affiliations:** ^1^ Department of Cardiology Hamad Medical Corporation Doha Qatar; ^2^ Department of Internal Medicine University of Toledo Medical Center Toledo Ohio USA; ^3^ Department of Internal Medicine Hamad Medical Corporation Doha Qatar; ^4^ Department of Internal Medicine MedStar Health Baltimore Maryland USA; ^5^ Department of Gastroenterology University of Toledo Medical Center Toledo Ohio USA

**Keywords:** acalculous cholecystitis, cardiogenic shock, case report, heart failure

## Abstract

Acute acalculous cholecystitis refers to inflammation of the gallbladder without the presence of gallstones or obstruction of the cystic duct. Heart failure is recognized for causing congestive hepatopathy and can lead to gallbladder swelling, often challenging to distinguish from acalculous cholecystitis. Here, we present a case of a patient whose symptoms initially resembled acalculous cholecystitis but were instead caused by acute severe heart failure and cardiogenic shock. Maintaining a broad differential diagnosis, including decompensated heart failure, is essential when evaluating cases resembling acalculous cholecystitis.

**Summary**

- Early recognition of congestive heart failure (CHF) presenting as cholecystalgia due to gallbladder edema is crucial.
- Maintaining a broad differential diagnosis, including decompensated HF, and using ultrasound imaging can help identify the underlying cause and guide appropriate treatment.

**Abbreviations**
CABG: coronary artery bypass grafting
CVP: central venous pressure
LAD: left anterior descending artery
LCx: left circumflex artery
LVEF: left ventricular ejection fraction
PCI: percutaneous coronary intervention
PCWP: pulmonary capillary wedge pressure
POBA: plain old balloon angioplasty
Pro‐BNP: pro‐brain natriuretic peptide
RUQ: right upper quadrant

## 1 Introduction

Acute cholecystitis is a common cause of acute abdominal pain, typically presenting with symptoms such as fever, abdominal pain, and jaundice. The most frequently encountered form of cholecystitis results from mechanical obstruction of the gallbladder outlet at the cystic duct, usually due to a gallstone [1]. The diagnosis of acute cholecystitis relies on radiological imaging techniques, including ultrasound (US), computed tomography (CT), and magnetic resonance imaging (MRI) [2]. In contrast, acute acalculous cholecystitis (AAC) is characterized by inflammation of the gallbladder without the presence of gallstones or obstruction of the cystic duct, often resulting from impaired gallbladder emptying or dysfunction. This form accounts for approximately 10% of all acute cholecystitis cases and 5%–10% of all cholecystitis instances [1]. AAC poses a significant diagnostic challenge due to its atypical clinical presentation, which often features a limited range of symptoms that can resemble those of cardiovascular disease [3]. This overlap can lead to underdiagnosis and misdiagnosis. Additionally, AAC typically follows a more fulminant course than calculous cholecystitis and is frequently associated with complications such as gangrene, perforation, and empyema [4, 5], resulting in substantial morbidity and mortality rates of up to 50% [6, 7]. Early diagnosis is essential for prompt treatment, as it can help prevent complications and improve survival rates.

Heart failure (HF) is a clinical syndrome commonly caused by structural or functional abnormalities of the heart, resulting in decreased cardiac output and congestion in other organs [8]. Acute congestive HF primarily manifests with signs of congestion and may also present with symptoms of organ hypoperfusion or cardiogenic shock [9]. The most frequently reported symptom is shortness of breath, while other common symptoms include chest pain, anorexia, and exertional fatigue. Anorexia in this context is often attributed to hepatic congestion, bowel edema, and decreased blood flow to the splanchnic circulation [8].

In most instances, the clinical manifestations of acute cholecystitis can be readily differentiated from those of congestive HF, given the diverse symptoms associated with each condition. However, in patients without a history of cardiovascular disease or those who do not fall into typical high‐risk categories for HF, there is a potential for misdiagnosis between the two conditions [10]. If a diagnosis is overlooked in such cases, it may result in delayed treatment, further exacerbating the patient's condition and necessitating increased attention from clinicians. In this report, we describe the case of a 69‐year‐old patient whose symptoms initially resembled AAC but were instead caused by acute severe heart failure and cardiogenic shock.

## 2 Case Presentation

### 2.1 Case History and Examination

A 69‐year‐old man of West African descent with a medical history of type 2 diabetes mellitus and hypertension presented with a 2‐day history of fever, chills, generalized body aches, right upper quadrant (RUQ) abdominal pain, nausea, and vomiting. On physical examination, he was afebrile but tachypneic, with blood pressure in the lower normal range, appeared unwell, and was deeply jaundiced. He had decreased breath sounds at the lung bases without crepitations. Additionally, he exhibited positive hepatojugular reflux, RUQ tenderness, and shifting dullness.

### 2.2 Investigations and Treatment

**FIGURE 1:**
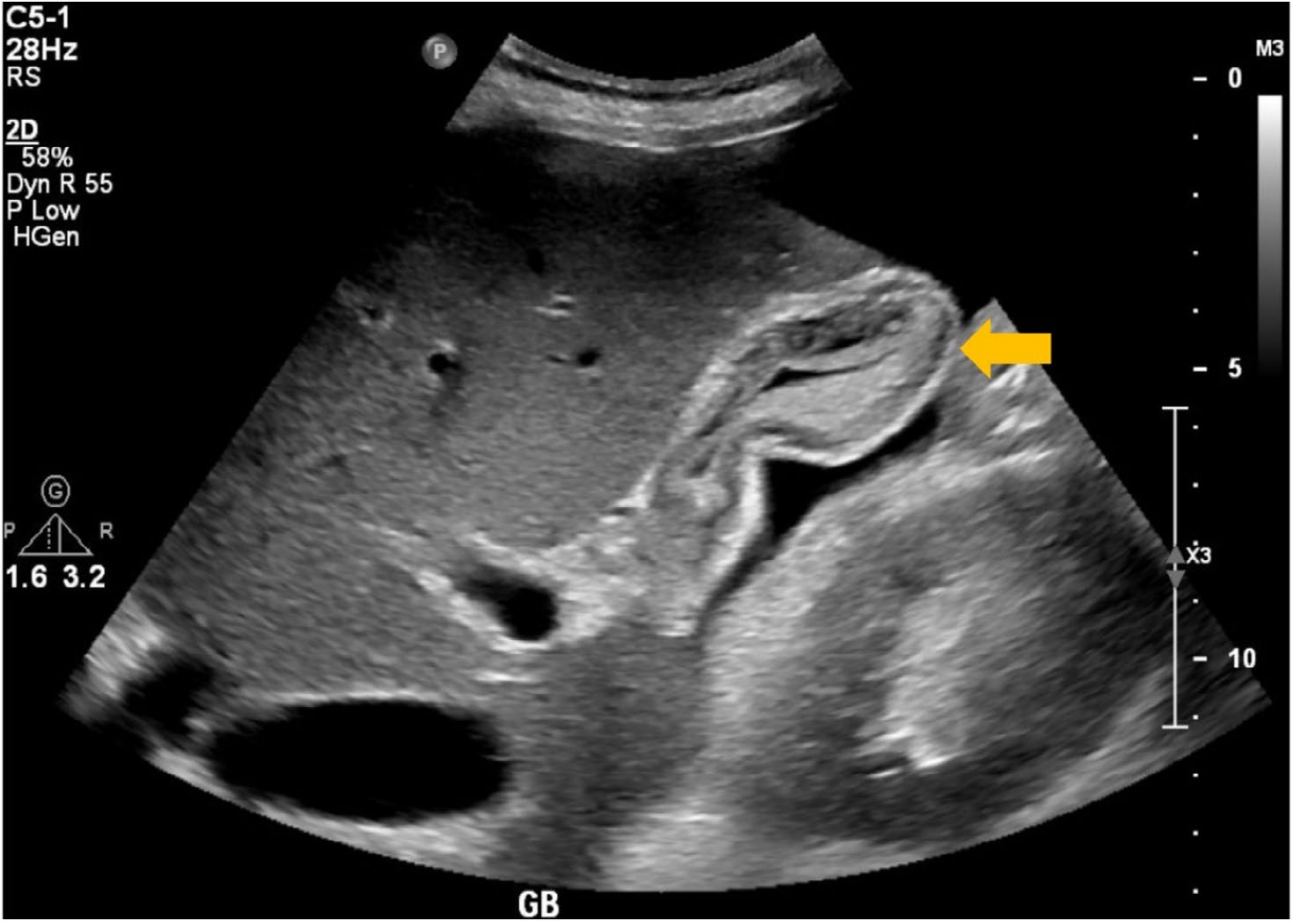
An ultrasound of the abdomen showing a thick and swollen gallbladder wall.

**FIGURE 2:**
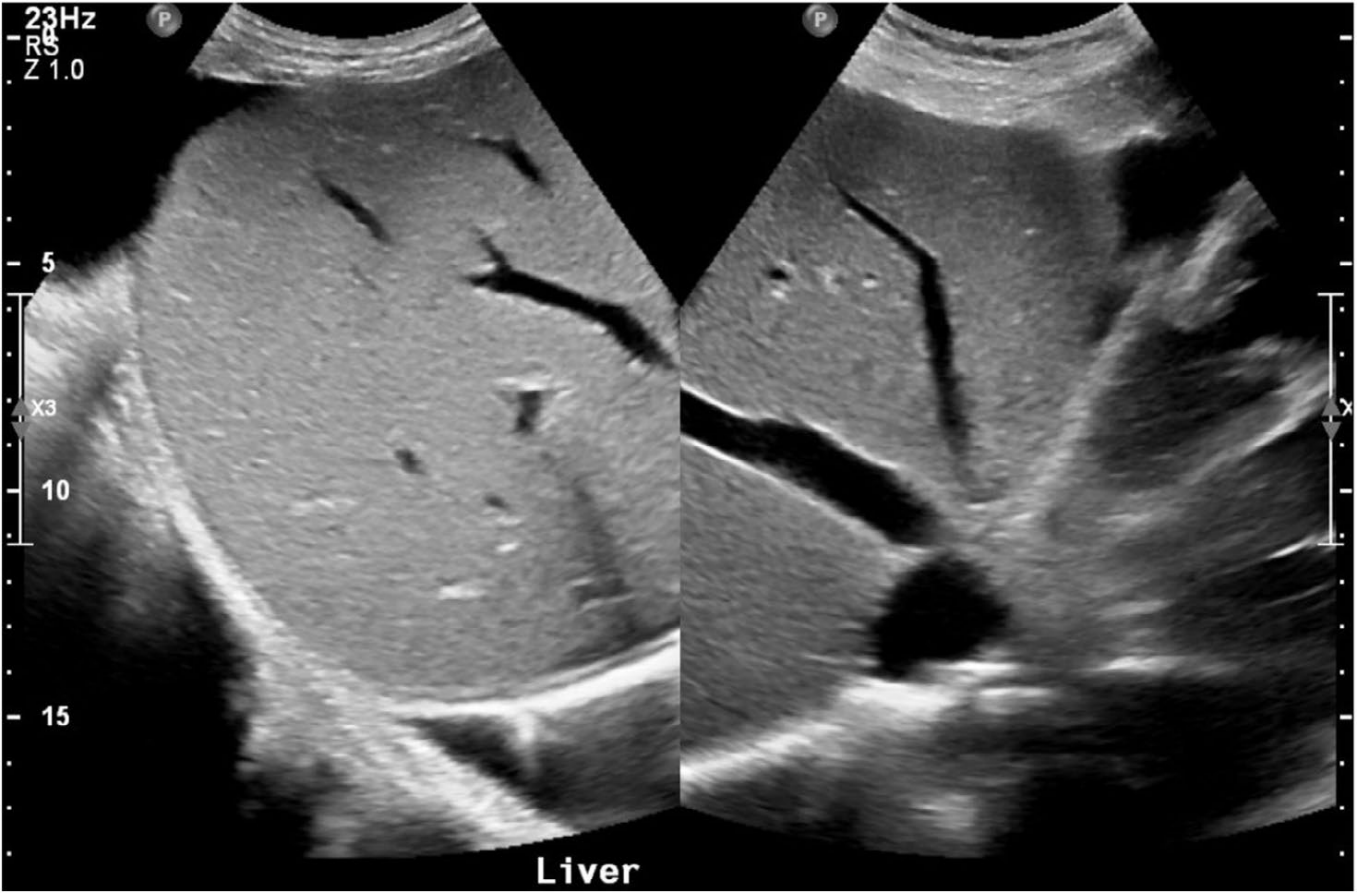
An ultrasound of the abdomen showing congestive hepatopathy with dilated hepatic veins and inferior vena cava.

Laboratory tests indicated deranged renal and liver functions with elevated procalcitonin, lactic acid, and Pro‐BNP (Table 1). Abdominal ultrasound revealed moderate ascites, congestive hepatopathy, and a thickened, edematous gallbladder wall without gallstones (Figures 1 and 2), findings that were initially interpreted as consistent with acalculous cholecystitis. He was treated with intravenous antibiotics and IV fluids, but his condition continued to deteriorate, and he became hypotensive.

**TABLE 1:** Initial laboratory test results.

Detail	Value w/Units	Normal range
CRP	18.7 mg/L	0.0–5.0
Lactic acid	3.0 mmol/L	0.5–2.2
Procalcitonin	3.34 ng/mL	—
Total bilirubin	56 μmol/L	0–21
Direct bilirubin	37 μmol/L	0–5
pH	7.307	7.320–7.420
White blood cell count	4.3 × 10^3^/μL	4.0–10.0
Hemoglobin	11.9 g/dL	13.0–17.0
Troponin‐T HS	26 ng/L	3–15
INR	3.3	—
APTT	31.4 s	25.1–36.5
Urea	19 mmol/L	2.5–7.8
Creatinine	268 μmol/L	62–106
Alkaline phosphatase	199 U/L	40–129
ALT	202 U/L	0–41
AST	283 U/L	0–40
NT pro‐BNP	16,088 pg/mL	—

Abbreviations: ALT, alanine aminotransferase; APTT, activated partial thromboplastin time; AST, aspartate aminotransferase; CRP, C‐reactive protein; INR, International Normalized Ratio; NT pro‐BNP, N‐terminal pro‐B‐type natriuretic peptide; Troponin‐T HS, high‐sensitivity troponin T.

An echocardiogram revealed remarkable biventricular and biatrial dilatation, a left ventricular ejection fraction (LVEF) of 25% with severe hypokinesia, grade 3 diastolic dysfunction, and severe tricuspid regurgitation. Estimated PCWP was 25 mmHg, and cardiac output was 3 L per minute (Video 1). The subsequent diagnosis was biventricular cardiogenic shock and secondary multisystem organ failure (encephalopathy, ischemic/congestive hepatopathy, and cardiorenal syndromes). The patient was transferred to the cardiac intensive care unit, where his central venous pressure (CVP) was 35 cm water. Norepinephrine, dobutamine, and furosemide infusions were administered, resulting in significant clinical and biochemical improvement (Table 2).

**VIDEO 1:** Echocardiography (a) parasternal long axis view and (b) apical four‐chamber view showing biventricular and biatrial dilatation with severe hypokinesia. Video content can be viewed at https://onlinelibrary.wiley.com/doi/10.1002/ccr3.70324

**TABLE 2:** Laboratory test results after stabilization.

Detail	Value w/Units	Normal range
Creatinine	108 μmol/L	62–106
ALT	46 U/L	0–41
AST	29 U/L	0–40
NT pro‐BNP	3437 pg/mL	—
INR	1.1	—
Total bilirubin	10 μmol/L	0–21

Abbreviations: ALT, alanine aminotransferase; APTT, activated partial thromboplastin time; AST, aspartate aminotransferase; CRP, C‐reactive protein; INR, International Normalized Ratio; NT pro‐BNP, N‐terminal pro‐B‐type natriuretic peptide; Troponin‐T HS, high‐sensitivity troponin T.

### 2.3 Outcome

After stabilization, angiography revealed three‐vessel disease, and he underwent successful PCIs to the mid LAD, POBA to D2, and PCI to D1 and LCx after refusing CABG. The patient was transferred to the ward, heart failure medications were optimized, and he was discharged in stable condition. He traveled back to his home country.

## 3 Discussion

Acute acalculous cholecystitis is closely linked to cardiovascular disease. Various studies indicate that AAC is associated with hypoperfusion states, such as acute myocardial infarction and acute HF, which can result in ischemic damage to the gallbladder [11, 12, 13, 14]. Similarly, a hypovolemic state resulting from the treatment of certain cardiovascular diseases may also contribute to the pathogenesis of AAC [3]. Regardless of the underlying etiology, the physiological consequences of AAC stem from visceral hypoperfusion, ischemia, reperfusion injury, and bile stasis [1].

Acute acalculous cholecystitis is often characterized by an atypical clinical onset and a limited range of symptoms that can overlap with those of comorbidities [3]. Confounding factors, such as the critical condition of patients, may result in negative or nonspecific laboratory findings during the early stages of the disease. These overlapping symptoms and laboratory data can complicate the differentiation of AAC from cardiovascular disease [15]. Both calculous and AAC can complicate the clinical presentation of ischemic cardiac events, heart failure, and stroke [16, 17]. However, the precise triggering mechanisms and pathobiological processes underlying the “cholecysto‐cardiac” link remain to be fully understood [18, 19].

In the emergency department, RUQ pain is a common symptom, with acute cholecystitis being a leading cause [20, 21]. Other prevalent causes include retrocecal appendicitis, acute hepatitis, and both acute and chronic pancreatitis [20]. Less frequently, RUQ pain may indicate cholecystalgia, which arises from gallbladder edema, typically secondary to various conditions, such as congestive HF, alcoholic and nonalcoholic liver cirrhosis, acute hepatitis, renal failure, and hypoproteinemia [22, 23, 24]. HF can result in hepatic congestion and/or isolated gallbladder edema, complicating the differentiation from acalculous cholecystitis [25]. The term “cholecystalgia” specifically refers to gallbladder pain in the absence of other RUQ causes, such as hepatic capsule distension due to congestive hepatopathy [22, 23, 24].

Furthermore, congestive HF may present with RUQ pain, and ultrasonographic findings can reveal an edematous gallbladder. This edema may occur secondary to increased portal venous pressure or ischemia resulting from cardiac instability, leading to a mistaken diagnosis of cholecystitis [19]. Notably, some of these changes may be transient and resolve with improvements in cardiac function, indicating that surgical intervention for cholecystitis may not be necessary [17]. Differentiating this condition from acute cholecystitis requires careful correlation of imaging findings with clinical and systemic parameters.

In our case, the absence of known cardiac issues initially suggested an acute condition. Although the patient did not meet all the diagnostic criteria outlined in the Tokyo Guidelines 2013 [26], the presence of systemic signs of inflammation, such as elevated inflammatory markers and localized RUQ tenderness, combined with the US finding of gallbladder wall thickening, prompted an initial diagnosis of AAC. This approach aimed to mitigate the risk of complications associated with delayed treatment, particularly given AAC's diagnostic challenges in patients with overlapping conditions. However, upon retrospective analysis, the US findings were more suggestive of gallbladder wall edema secondary to systemic congestion from decompensated HF, especially after the result of echocardiography. The absence of critical imaging findings for acute cholecystitis, such as gallbladder distension or pericholecystic fluid, highlights the diagnostic challenge in distinguishing AAC from gallbladder changes due to congestive states.

The constellation of the patient's older age, history of cardiac risk factors, such as hypertension and diabetes, and the presence of hypotension, abdominal distension with fluid accumulation, and swelling in both lower limbs raised suspicion for a cardiac etiology underlying the symptoms. Decompensated HF can impair myocardial function, leading to shock and subsequent damage to the liver and gallbladder due to congestion and ischemia. The patient's hepatic and cardiac function improved with the administration of intravenous inotropic agents and diuretics. Similarly, two cases reported by Yu and colleagues described young males (ages 26 and 39) who were misdiagnosed with acute cholecystitis but experienced significant relief of symptoms after receiving treatment with diuretics and cardiac glycosides [10].

In acute HF, RUQ pain can result from hepatic congestion, hepatobiliary stasis, or ascites, all of which may mimic the symptoms of acute cholecystitis. These symptoms are often exacerbated by mesenteric hypoperfusion, leading to cramp‐like abdominal pain. Radiologically, gallbladder wall thickening in HF is typically caused by venous congestion and systemic inflammation rather than primary gallbladder pathology, highlighting the nonspecific nature of this finding. Additionally, elevated inflammatory markers in HF are often a result of systemic cytokine release or mesenteric hypoperfusion‐induced endotoxemia, further complicating the differentiation from true inflammatory conditions.

This case prompted the diagnosis of HF in a patient who presented with only abdominal discomfort and lacked typical signs and symptoms of congestive HF. A thorough investigation resulted in an accurate diagnosis, ultimately preventing an unnecessary and potentially risky laparoscopic cholecystectomy. Thus, it is essential for physicians to maintain a high index of suspicion in order to accurately diagnose such cases and avoid unnecessary procedures.

## 4 Conclusion

This case illustrates an atypical presentation of congestive HF. Cholecystalgia resulting from gallbladder edema is a rare condition. It is essential to maintain a broad differential diagnosis, including decompensated HF, when evaluating cases that resemble acalculous cholecystitis. Early recognition of the symptoms, along with diagnosis via US imaging, allows physicians to identify the underlying cause and implement appropriate treatment.

## Author Contributions

**Mhd Baraa Habib:** conceptualization, data curation, methodology, supervision, writing – review and editing. **Maram Albandak:** supervision, writing – original draft, writing – review and editing. **Mhd Husam Osman:** conceptualization, writing – review and editing. **Shahem Abbarh:** data curation, writing – original draft, writing – review and editing. **Bisher Sawaf:** conceptualization, supervision, writing – original draft, writing – review and editing. **Yaseen Alastal:** supervision, writing – review and editing. **Abdulrahman Arabi:** conceptualization, supervision, writing – review and editing.

## Consent

Written informed consent was obtained from the patient for anonymized patient information to be published in this article.

## Conflicts of Interest

The authors declare no conflicts of interest.

## Data Availability

The datasets generated during and/or analyzed during the current study are available from the corresponding author upon reasonable request.

## References

[1] M. W. Jones and T. Ferguson , “Acalculous Cholecystitis,” in StatPearls (StatPearls Publishing, 2024). 29083717

[2] N. K. Wee , W. S. C. Cheong , and H. M. Low , “CT and MRI Findings of Acute Calculous Cholecystitis and Its Complications in Singapore: A Pictorial Review,” Medical Journal of Malaysia 76, no. 5 (2021): 706–713. 34508378

[3] M. Saragò , D. Fiore , S. De Rosa , et al., “Acute Acalculous Cholecystitis and Cardiovascular Disease, Which Came First? After Two Hundred Years Still the Classic Chicken and Eggs Debate: A Review of Literature,” Annals of Medicine and Surgery 78 (2012): 103668. 10.1016/j.amsu.2022.103668 PMC9206910 35734727

[4] T. Inoue and Y. Mishima , “Postoperative Acute Cholecystitis: A Collective Review of 494 Cases in Japan,” Japanese Journal of Surgery 18, no. 1 (1988): 35–42. 3290556 10.1007/BF02470844

[5] E. A. Deitch and J. M. Engel , “Ultrasonic Detection of Acute Cholecystitis With Pericholecystic Abscesses,” American Surgeon 47, no. 5 (1981): 211–214. 7015938

[6] S. Kalliafas , D. W. Ziegler , L. Flancbaum , and P. S. Choban , “Acute Acalculous Cholecystitis: Incidence, Risk Factors, Diagnosis, and Outcome,” American Surgeon 64, no. 5 (1998): 471–475. 9585788

[7] A. J. Wang , “Clinical Predictors of Severe Gallbladder Complications in Acute Acalculous Cholecystitis,” World Journal of Gastroenterology 9, no. 12 (2003): 2821. 14669342 10.3748/wjg.v9.i12.2821 PMC4612061

[8] A. Malik , D. Brito , S. Vaqar , and L. Chhabra , “Congestive Heart Failure,” in StatPearls (StatPearls Publishing, 2024).

[9] A. H. Behnoush , A. Khalaji , N. Naderi , H. Ashraf , and S. von Haehling , “ACC/AHA/HFSA 2022 and ESC 2021 Guidelines on Heart Failure Comparison,” ESC Heart Failure 10, no. 3 (2023): 1531–1544. 36460629 10.1002/ehf2.14255 PMC10192289

[10] Q. Yu and W. Lai , “Heart Failure Misdiagnosed as Acute Cholecystitis: A Case Report,” Journal of Medical Case Reports 18, no. 1 (2024): 497. 39407349 10.1186/s13256-024-04829-0 PMC11481506

[11] J. L. Huffman and S. Schenker , “Acute Acalculous Cholecystitis: A Review,” Clinical Gastroenterology and Hepatology 8, no. 1 (2010): 15–22. 19747982 10.1016/j.cgh.2009.08.034

[12] H. Doran , O. Mihalache , F. Bobircă , C. Bugă , and T. Pătraşcu , “Acute Acalculous Cholecystitis—Difficulties of Diagnosis and Treatment,” Chirurgia 105, no. 4 (1990): 465–468. 20941966

[13] P. Ortega Deballon and A. de Lorenzo‐Cáceres , “Acute Acalculous Cholecystitis and Acute Myocardial Infarct,” Revista Clínica Española 197, no. 6 (1997): 464. 9304143

[14] K. Kubota , Y. Abe , M. Inamori , et al., “Percutaneous Transhepatic Gallbladder Stenting for Recurrent Acute Acalculous Cholecystitis After Failed Endoscopic Attempt,” Journal of Hepato‐Biliary‐Pancreatic Surgery 12, no. 4 (2005): 286–289. 16133694 10.1007/s00534-005-0989-9

[15] M. Ozeki , Y. Takeda , H. Morita , et al., “Acute Cholecystitis Mimicking or Accompanying Cardiovascular Disease Among Japanese Patients Hospitalized in a Cardiology Department,” BMC Research Notes 8, no. 1 (2015): 805. 26686987 10.1186/s13104-015-1790-8 PMC4684918

[16] Y. Kuroi , D. Imazato , K. Yamazaki , and H. Kasuya , “Acute Cholecystitis in Patients With Stroke,” Neurology India 67, no. 2 (2019): 439–441. 31085856 10.4103/0028-3886.258055

[17] M. Nagappa and A. Taly , “Cholecysto‐Cardiac Link: The Heart of the Matter,” Neurology India 67, no. 2 (2019): 391–392. 31085842 10.4103/0028-3886.258015

[18] S. Haleem , M. M. Ansari , T. Z. Khan , and M. H. Beg , “Cholecysto‐Cardiac Link,” Indian Journal of Medical Research 94 (1991): 47–49. 2071183

[19] Y. AlShehri , H. ElShafei , A. Dajani , M. AlArifi , and F. AlAradi , “Fatal Reversal of the Cholecystocardiac Link,” Journal of Pediatric Surgery Case Reports 32 (2018): 79–81.

[20] J. Avegno and M. Carlisle , “Evaluating the Patient With Right Upper Quadrant Abdominal Pain,” Emergency Medicine Clinics of North America 34, no. 2 (2016): 211–228. 27133241 10.1016/j.emc.2015.12.011

[21] J. R. Gallaher and A. Charles , “Acute Cholecystitis: A Review,” Journal of the American Medical Association 327, no. 10 (2022): 965. 35258527 10.1001/jama.2022.2350

[22] C. N. Desautels , D. M. Tierney , F. Rossi , and T. K. Rosborough , “Case Report: An Unrecognized Etiology of Transient Gallbladder Pain in Heart Failure Diagnosed With Internist‐Performed Point‐of‐Care Ultrasound,” Critical Ultrasound Journal 7, no. 1 (2015): 2. 25852843 10.1186/s13089-014-0019-8 PMC4384720

[23] M. Murthi , A. R. Abusaleem , Z. Haque , and S. U. Banskota , “Heart Failure Exacerbation Presenting as CHOLECYSTALGIA,” Journal of the American College of Cardiology 77, no. 18 (2021): 2141.

[24] A. Finazzi and R. Cosentini , “Cholecystalgia: An Atypical Presentation of Congestive Heart Failure in a New Diagnosis of Alcohol‐Induced Cardiomyopathy,” Annals of Internal Medicine: Clinical Cases 2, no. 1 (2023): e220857.

[25] M. Tana , C. Tana , G. Cocco , G. Iannetti , M. Romano , and C. Schiavone , “Acute Acalculous Cholecystitis and Cardiovascular Disease: A Land of Confusion,” Journal of Ultrasound 18, no. 4 (2015): 317–320. 26550069 10.1007/s40477-015-0176-z PMC4630273

[26] M. Yokoe , T. Takada , S. M. Strasberg , et al., “New Diagnostic Criteria and Severity Assessment of Acute Cholecystitis in Revised Tokyo Guidelines,” Journal of Hepato‐Biliary‐Pancreatic Sciences 19, no. 5 (2012): 578–585. 22872303 10.1007/s00534-012-0548-0 PMC3429769

